# Complete genome sequence of *Parvibaculum lavamentivorans* type strain (DS-1^T^)

**DOI:** 10.4056/sigs.2215005

**Published:** 2011-12-22

**Authors:** David Schleheck, Michael Weiss, Sam Pitluck, David Bruce, Miriam L. Land, Shunsheng Han, Elizabeth Saunders, Roxanne Tapia, Chris Detter, Thomas Brettin, James Han, Tanja Woyke, Lynne Goodwin, Len Pennacchio, Matt Nolan, Alasdair M. Cook, Staffan Kjelleberg, Torsten Thomas

**Affiliations:** 1 Department of Biological Sciences and Research School Chemical Biology, University of Konstanz, Germany; 2 DOE Joint Genome Institute, Walnut Creek, California, USA; 3 Los Alamos National Laboratory, Bioscience Division, Los Alamos, New Mexico, USA; 4 Oak Ridge National Laboratory, Oak Ridge, Tennessee, USA; 5 Centre for Marine Bio-Innovation and School of Biotechnology and Biomolecular Science, University of New South Wales, Sydney, Australia

**Keywords:** *Parvibaculum lavamentivorans* DS-1, aerobic, Gram-negative, *Rhodobiaceae*, surfactant biodegradation

## Abstract

*Parvibaculum lavamentivorans* DS-1^T^ is the type species of the novel genus *Parvibaculum* in the novel family *Rhodobiaceae* (formerly *Phyllobacteriaceae*) of the order *Rhizobiales* of *Alphaproteobacteria*. Strain DS-1^T^ is a non-pigmented, aerobic, heterotrophic bacterium and represents the first tier member of environmentally important bacterial communities that catalyze the complete degradation of synthetic laundry surfactants. Here we describe the features of this organism, together with the complete genome sequence and annotation. The 3,914,745 bp long genome with its predicted 3,654 protein coding genes is the first completed genome sequence of the genus *Parvibaculum*, and the first genome sequence of a representative of the family *Rhodobiaceae*.

## Introduction

*Parvibaculum lavamentivorans* strain DS-1^T^ (DSM13023 = NCIMB13966) was isolated for its ability to degrade linear alkylbenzenesulfonate (LAS), a major laundry surfactant with a world-wide use of 2.5 million tons per annum [1]. Strain DS-1^T^ was difficult to isolate, is difficult to cultivate, and represents a novel genus in the *Alphaproteobacteria* [2,3]. Strain DS-1 catalyzes not only the degradation of LAS, but also of 16 other commercially important anionic and non-ionic surfactants (hence the species name *lavamentivorans* = consuming [chemicals] used for washing [3]). The initial degradation as catalyzed by strain DS-1^T^ involves the activation and shortening of the alkyl-chain of the surfactant molecules, and the excretion of short-chain degradation intermediates. These intermediates are then completely utilized by other bacteria in the community [4,5]. *P. lavamentivorans* DS-1^T^ is therefore an example of a first tier member of a two-step process that mineralizes environmentally important surfactants.

Other representatives of the novel genus *Parvibaculum* have been recently isolated. *Parvibaculum* sp. strain  JP-57 was isolated from seawater [6] and is also difficult to cultivate [3]. *Parvibaculum indicum* sp. nov. was also isolated from seawater, via an enrichment culture that degraded polycyclic aromatic hydrocarbons (PAH) and crude oil [7]. Another *Parvibaculum* sp. strain was isolated from a PAH-degrading enrichment culture, using river sediment as inoculum [8]. *Parvibaculum* species were also reported in a study on marine alkane-degrading bacteria [9]. *Parvibaculum* species are frequently detected by cultivation-independent methods, predominantly in habitats or settings with hydrocarbon degradation. These include a bacterial community on marine rocks polluted with diesel oil [10], a bacterial community from diesel-contaminated soil [11], a petroleum-degrading bacterial community from seawater [12], an oil-degrading cyanobacterial community [13] and biofilm communities in pipes of a district heating system [14]. *Parvibaculum* species have also been detected in denitrifying, linear-nonylphenol (NP) degrading enrichment cultures from NP-polluted river sediment [15] and in groundwater that had been contaminated by linear alkyl benzenes (LABs; non-sulfonated LAS] [16]. Additionally, *Parvibaculum* species were detected in biofilms that degraded polychlorinated biphenyls (PCBs) using pristine soil as inoculum [17], and in a PAH-degrading bacterial community from deep-sea sediment of the West Pacific [18]. Finally, *Parvibaculum* species were detected in an autotrophic Fe(II)-oxidizing, nitrate-reducing enrichment culture [19], as well as in Tunisian geothermal springs [20]. The widespread occurrence of *Parvibaculum* species in habitats or settings related to hydrocarbon degradation implies an important function and role of these organisms in environmental biodegradation, despite their attribute as being difficult to cultivate in a laboratory.

Here we present a summary classification and a set of features for *P. lavamentivorans* DS-1^T^, together with the description of a complete genome sequence and annotation. The genome sequencing and analysis was part of the Microbial Genome Program of the DOE Joint Genome Institute.

## Classification and features

**Figure 1:**
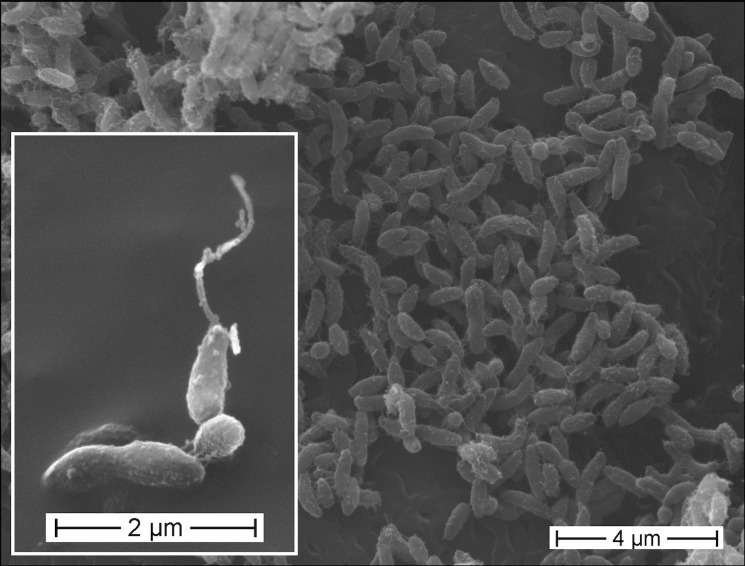
Scanning electron micrograph of *P. lavamentivorans* DS-1^T^. Cells derived from a liquid culture that grew in acetate/mineral salts medium.

*P. lavamentivorans* DS-1^T^ is a Gram-negative, non-pigmented, very small (approx. 1.0 × 0.2 µm), slightly curved rod-shaped bacterium that can be motile by means of a polar flagellum (Figure 1, Table 1). Strain DS-1^T^ grows very slowly on complex medium (e.g. on LB- or peptone-agar plates) and forms pinpoint colonies only after more than two weeks of incubation. The organism can be quickly overgrown by other organisms. Larger colonies are obtained when the complex medium is supplemented with a surfactant, e.g. Tween 20 (see DSM-medium 884 [29]) or LAS [3]. When cultivated in liquid culture with mineral-salts medium, strain DS-1^T^ grows within one week with the single carbon sources acetate, ethanol, or succinate, or alkanes, alkanols and alkanoates (C_8_ - C_16_); no sugars tested were utilized [3].

**Table 1:** Classification and general features of *Parvibaculum lavamentivorans* DS-1^T^.

**MIGS ID**	**Property**	**Term**	**Evidence code**^a^
	Current classification	Domain *Bacteria*	TAS [21]
	Phylum	*Proteobacteria*	TAS [22]
	Class	*Alphaproteobacteria*	TAS [23,24]
	Order	*Rhizobiales*	TAS [23,25]
	Family	*Rhodobiaceae*	TAS [23,26]
	Genus	*Parvibaculum*	TAS [3]
	Species	*Parvibaculum lavamentivorans*	TAS [3]
		Type strain DS-1	
	Gram stain	negative	TAS [3]
	Cell shape	small rod	TAS [3]
	Motility	motile	TAS [3]
	Sporulation	non-sporulating	TAS [3]
	Temperature range	mesophile	TAS [3]
	Optimum temperature	30 ºC	TAS [3]
	Carbon source	acetate, ethanol, pyruvate, succinate, alkanes (C_8_ – C_16_), various anionic and non-ionic surfactants	TAS [2,3,5,7]
	Energy source	chemoorganotroph	TAS [3]
	Terminal electron receptor	molecular oxygen	TAS [3]
MIGS-6	Habitat	aerobic habitat	TAS [2,27]
MIGS-6.3	Salinity	0 – 3% NaCl	TAS [3]
MIGS-22	Oxygen requirement	aerobic	TAS [3]
MIGS-15	Biotic relationship	free-living	TAS [3]
MIGS-14	Pathogenicity	none	TAS [3]
MIGS-4	Geographic location	isolated from a surfactant-degrading laboratory trickling filter that was inoculated with sludge of an industrial sewage treatment plant in Ludwigshafen, Germany	TAS [2]
MIGS-5	Sample collection time	1999	TAS [2]
MIGS-4.1	Latitude	49.48	TAS [2]
MIGS-4.2	Longitude	8.44	TAS [2]
MIGS-4.3	Depth		
MIGS-4.4	Altitude	96 m	TAS [2]

a) Evidence codes - IDA: Inferred from Direct Assay; TAS: Traceable Author Statement (i.e., a direct report exists in the literature); NAS: Non-traceable Author Statement (i.e., not directly observed for the living, isolated sample, but based on a generally accepted property for the species, or anecdotal evidence). These evidence codes are from of the Gene Ontology project [28].

To allow for growth in liquid culture with most of the 16 different surfactants at high concentrations (e.g. for LAS, >1 mM; see [3].), the culture fluid needs to be supplemented with a solid surface, e.g. polyester fleece or glass fibers [2,3]. The additional solid surface is believed to support biofilm formation, especially in the early growth phase when the surfactant concentration is high, and the organism grows as single, suspended cells (non-motile) during the later growth phase. Growth with a non-membrane toxic substrate (e.g. acetate) is independent of a solid surface, and constitutes suspended, single cells (motile). We presume that the biofilm formation by strain DS-1^T^ is a protective response to the exposure to membrane-solubilizing agents (cf. [30]).

**Figure 2:**
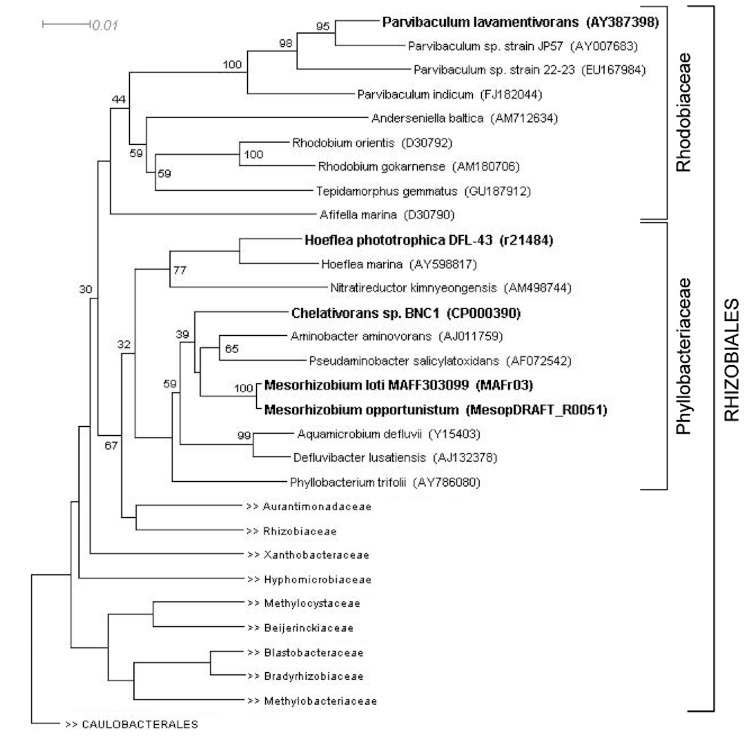
Phylogenetic tree of 16S rRNA gene sequences showing the position of *P. lavamentivorans* DS-1^T^ relative to other type strains within the families *Rhodobiaceae*, *Phyllobacteriaceae* and other families in the order *Rhizobiales* (see the text). Strains within the *Rhodobiaceae* and *Phyllobacteriaceae* shown in bold have genome projects underway or completed. The corresponding 16S rRNA gene accession numbers (or draft genome sequence identifiers) are indicated. The sequences were aligned using the GreenGenes NAST alignment tool [37]; neighbor-joining tree building and visualization involved the CLUSTAL and DENDROSCOPE software [38]. *Caulobacterales* sequences were used as outgroup. Bootstrap values >30 % are indicated; bar, 0.01 substitutions per nucleotide position.

Based on the 16S rRNA gene sequence, strain DS-1^T^ was described as the novel genus *Parvibaculum*, which was originally placed in the family *Phyllobacteriaceae* within the order *Rhizobiales* of *Alphaproteobacteria* [3,31]. The nearest well-described organism to strain DS-1^T^ is *Afifella marina* (formerly *Rhodobium marinum*) (92% 16S rRNA gene sequence identity), a photosynthetic purple, non-sulfur bacterium. The genus *Rhodobium* was later re-classified as a member of the novel family *Rhodobiaceae* [26,32], together with two novel genera of other photosynthetic purple non-sulfur bacteria (*Afifella* and *Roseospirillum*), as well as with two novel genera of heterotrophic aerobic bacteria, represented by the red-pigmented *Anderseniella baltica* (gen. nov., sp. nov.) [33,34] and non-pigmented *Tepidamorphus gemmatus* (gen. nov., sp. nov.) [35,36]. A phylogenetic tree (Figure 2) was constructed with the 16S rRNA gene sequence of *P. lavamentivorans* DS-1^T^ and that of (i) other isolated *Parvibaculum* strains, (ii) representatives of other genera within the family *Rhodobiaceae*, (iii) representatives of the genera in the family *Phyllobacteriaceae*, as well as, (iv) representatives of other families within the order *Rhizobiales*. The phylogenetic tree shows now the placement of *Parvibaculum* species within the family *Rhodobiaceae*, and that the *Parvibaculum* sequences clustered as a distinct evolutionary lineage within this family (Figure 2). This classification of *Parvibaculum* has been adopted in the Ribosomal Database Project (RDP) and SILVA rRNA Database Project, but not in the GreenGenes database. The family *Rhodobiaceae* has also not been included in the NCBI-taxonomy, IMG-taxonomy, and GOLD databases.

Currently, 360 genome sequences of members of the order *Rhizobiales* of *Alphaproteobacteria* have been made available (GOLD database; August 2011), and within the family *Phyllobacteriaceae* there are 21 genome sequences available (*Chelativorans* sp. BNC1, *Hoeflea phototrophica* DFL-43, and 18 *Mesorhizobium* strains). No genome sequences currently exist for a representative of the novel family *Rhodobiaceae*, except of the genome of *P. lavamentivorans* DS-1^T^.

### Chemotaxonomy

Examination of the respiratory lipoquinone composition of strain DS-1^T^ showed that ubiquinones are the sole respiratory quinones present, and the major lipoquinone is ubiquinone 11 (Q11) [3]. The fatty acids of *P. lavamentivorans* are straight chain saturated and unsaturated, as well as ester- and amide-linked hydroxy-fatty acids, in membrane fractions [3]. The major polar lipids are phosphatidyl glycerol, diphosphatidyl glycerol, phosphatidyl ethanolamine, phosphatidyl choline, and two, unidentified aminolipids; the presence of the two additional aminolipids appears to be distinctive of the organism [3]. The G+C content of the DNA was determined to be 64% [3], which corresponds well to the G+C content observed for the complete genome sequence (see below).

## Genome sequencing information

### Genome project history

The genome was selected for sequencing as part of the U.S. Department of Energy - Microbial Genome Program 2006. The DNA sample was submitted in April 2006 and the initial sequencing phase was completed in October 2006. The genome finishing and assembly phase were completed in June 2007, and presented for public access on December 2007; a modified version was presented in February 2011. Table 2 presents the project information and its association with MIGS version 2.0 compliance [39].

**Table 2:** Project information

**MIGS ID**	**Property**	**Term**
MIGS-31	Finishing quality	Finished
MIGS-28	Libraries used	3.5 kb, 9 kb and 37 kb DNA libraries
MIGS-29	Sequencing platforms	Sanger
MIGS-31.2	Sequence coverage	16×
MIGS-30	Assemblers	Phred/Phrap/Consed
MIGS-32	Gene calling method	Glimmer/Criteria
	Genbank ID	17639
	Genbank Date of Release	July 31, 2007
	GOLD ID	Gc00631
MIGS-13	Source material identifier	DSM 13023 = NCIMB 13966
	Project relevance	Biodegradation, biotechnological

### Growth conditions and DNA isolation

*P. lavamentivorans* DS-1^T^ was grown on LB agar plates (2 weeks) and pinpoint colonies were transferred into selective medium (1 mM LAS/minimal salts medium; with glass-fiber supplement, 5-ml scale [3]). This culture was sub-cultivated to larger scale (100-ml and 1-liter scale) in 30 mM acetate/minimal salts medium; cell pellets were stored frozen until DNA preparation. DNA was prepared following the JGI DNA Isolation Bacterial CTAB Protocol [40].

### Genome sequencing and assembly

The genome of *P. lavamentivorans* DS-1^T^ was sequenced at the Joint Genome Institute (JGI) using a combination of 3.5 kb, 9 kb and 37 kb DNA libraries. All general aspects of library construction and sequencing performed at the JGI can be found at the JGI website [41]. Draft assemblies were based on 76,870 reads. Combined, the reads from all three libraries provided 16-fold coverage of the genome. The Phred/Phrap/Consed software package [42] was used for sequence assembly and quality assessment [43-45]. After the shotgun stage, reads were assembled with parallel phrap (High Performance Software, LLC). Possible mis-assemblies were corrected with Dupfinisher [46], PCR amplification, or transposon bombing of bridging clones (Epicentre Biotechnologies, Madison, WI, USA). Gaps between contigs were closed by editing in Consed, custom primer walk or PCR amplification (Roche Applied Science, Indianapolis, IN, USA). A total of 24 primer walk reactions were necessary to close gaps and to raise the quality of the finished sequence. The completed genome assembly contains 76,885 reads, achieving an average of  16-fold sequence coverage per base with an error rate less than 5 in 100,000.

### Genome annotation

Genes were identified using a combination of Critica [47] and Glimmer [48] as part of the genome annotation pipeline at Oak Ridge National Laboratory (ORNL), Oak Ridge, TN, USA, followed by a round of manual curation. The predicted CDSs were translated and used to search the National Center for Biotechnology Information (NCBI) non-redundant database, UniProt, TIGRFam, Pfam, PRIAM, KEGG, COG, and InterPro databases; miscellaneous features were predicted using TMHMM [49] and signalP [50]. These data sources were combined to assert a product description for each predicted protein. The tRNAScanSE tool [51] was used to find tRNA genes, whereas ribosomal RNAs were found by using BLASTn against the ribosomal RNA databases. The RNA components of the protein secretion complex and the RNaseP were identified by searching the genome for the corresponding Rfam profiles using INFERNAL [52]. Additional gene prediction analysis and manual functional annotation was performed within the Integrated Microbial Genomes (IMG) platform [41] developed by the Joint Genome Institute, Walnut Creek, CA, USA [53].

## Genome properties

**Figure 3:**
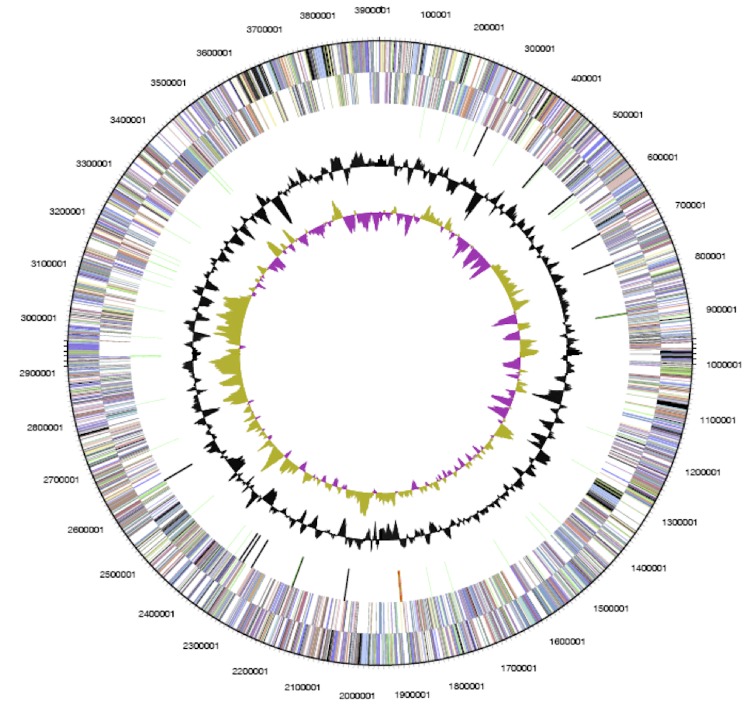
Graphical circular map of the genome of *P. lavamentivorans* DS-1^T^. From outside to center: Genes on forward strand (color by COG categories), genes on reverse strand (color by COG categories), RNA genes (tRNA, green; rRNA, red; other RNAs, black), GC content, GC skew.

The genome of *P. lavamentivorans* DS-1^T^ comprises one circular chromosome of 3,914,745 bp (62.33% GC content) (Figure 3), for which a total number of 3,714 genes were predicted. Of these predicted genes, 3,654 are protein-coding genes, and 2,723 of the protein-coding genes were assigned to a putative function and the remaining annotated as hypothetical proteins; 18 pseudogenes were also identified. A total of 60 RNA genes and one rRNA operon are predicted; the latter is reflective of the slow growth of *P. lavamentivorans* DS-1^T^ [54,55]. Furthermore, one Clustered Regularly Interspaced Short Palindromic Repeats element (CRISPR) including associated protein genes were predicted. The properties and the statistics of the genome are summarized in Table 3, and the distribution of genes into COGs functional categories is presented in Table 4.

**Table 3:** Nucleotide and gene count levels of the genome of *P. lavamentivorans* DS-1^T^

**Attribute**	**Value**	**% of total^a^**
Genome size (bp)	3,914,745	100
DNA coding region (bp)	3,535,064	90.30
G+C content (bp)	2,439,986	62.33
Number of replicons	1	
Extrachromosomal elements	0	
Total genes	3,714	100
RNA genes	60	1.62
rRNA operons	1	
Protein-coding genes	3,654	98.38
Pseudo genes	18	0.48
Genes with function prediction	2,723	73.32
Genes in paralog clusters	620	16.69
Genes assigned to COGs	2,904	78.19
Genes assigned to Pfam domains	3,054	82.23
Genes with signal peptides	717	19.31
Genes connected to KEGG pathways	1,085	29.21
Genes with transporter classification	430	11.58
Genes with transmembrane helices	782	21.06
CRISPR count	1	% of total^a^

^a^) The total is based on either the size of the genome in base pairs or the total number of protein coding genes in the annotated genome.

**Table 4:** Number of genes associated with the general COG functional categories in *P. lavamentivorans* DS-1^T^

**Code**	**Value**	**%age**	**Description**
J	163	5.07	Translation, ribosomal structure and biogenesis
A	1	0.0	RNA processing and modification
K	243	7.0	Transcription
L	137	3.9	Replication, recombination and repair
B	1	0.0	Chromatin structure and dynamics
D	25	0.7	Cell cycle control, mitosis and meiosis
Y	0	0.0	Nuclear structure
Z	0	0	Cytoskeleton
W	0	0	Extracellular structures
V	85	2.4	Defense mechanisms
T	118	3.4	Signal transduction mechanisms
M	131	3.8	Cell wall/membrane biogenesis
N	6	0.2	Cell motility
U	39	1.1	Intracellular trafficking and secretion
O	77	2.2	Posttranslational modification, protein turnover, chaperones
C	153	4.4	Energy production and conversion
G	294	8.4	Carbohydrate transport and metabolism
E	214	6.1	Amino acid transport and metabolism
F	79	2.3	Nucleotide transport and metabolism
H	110	3.2	Coenzyme transport and metabolism
I	73	2.1	Lipid transport and metabolism
P	152	4.4	Inorganic ion transport and metabolism
Q	30	0.9	Secondary metabolites biosynthesis, transport and catabolism
R	318	9.1	General function prediction only
S	200	5.7	Function unknown
-	1082	31.0	Not in COGs

## Metabolic features

The genome of *P. lavamentivorans* encodes complete pathways for synthesis of all proteinogenic amino acids and essential co-factors, and the central metabolism is represented by a complete pathway for the citrate cycle, glycolysis/gluconeogenesis, and the non-oxidative branch of the pentose-phosphate pathway; no candidate genes for the oxidative branch of the pentose-phosphate pathway or for the Entner–Doudoroff pathway are predicted.

*P. lavamentivorans* DS-1^T^ does not grow on D-glucose, D-fructose, maltose, D-mannitol, D-mannose, and  N-acetylglucosamine [3,7], and there are no valid candidate genes predicted in the genome for ATP-dependent sugar uptake systems or for D-glucose uptake via a phosphotransferase system. Similarly, no valid candidate genes were predicted for ATP-dependent amino-acid and di/oligo-peptide transport systems or for other amino-acid/peptide transporters, which reflects the poor growth of strain DS-1^T^ in complex medium (LB-medium).

For the assimilation of acetyl-CoA from the degradation of alkanes and surfactants [2,3,5], or during growth with acetate, the genome of *P. lavamentivorans* encodes the glyoxylate cycle (isocitrate lyase, Plav_0592; malate synthase, Plav_0593) to generate succinate for the synthesis of carbohydrates. The genome also encodes the complete ethyl-malonyl-CoA pathway to assimilate acetate [56]. This observation, i.e. glyoxylate cycle and ethyl-malonyl-CoA pathway in the same organism, has been made before [57], and these two pathways in  *P. lavamentivorans* DS-1^T^ might be differentially expressed under varying environmental conditions.

For the degradation of alkanes and surfactants through abstraction of acetyl-CoA [54], the genome contains a wealth of candidate genes for the entry into alkyl-chain degradation (*omega*-oxygenation to activate the chain) supplemented by a variety of genes predicted for *omega*-oxidations (to generate the corresponding fatty-acids) and fatty-acid *beta*-oxidations (to excise acetyl-CoA units). We are currently exploring this high abundance of genes for alkane/alkyl-utilization in strain DS-1^T^ by transcriptional and translational analysis [unpublished]. For example, at least nine cytochrome-P450 (CYP) alkane monooxygenase (COG2124), 44 alcohol dehydrogenase (COG1028), 11 aldehyde dehydrogenase (COG1012), 20 acyl-CoA synthetase (COG0318), 40 acyl-CoA dehydrogenase (COG1960), 31 enoyl-CoA hydratase (COG1024), 14 acyl-CoA acetyl-transferase (COG0183), six thioesterase (COG0824), and 17 putative long-chain acyl-CoA thioester hydrolase (PF03061) candidate genes are predicted in the genome.

Other predicted oxygenase genes comprise three putative Baeyer-Villiger-type FAD-binding monooxygenase genes (COG2072). Cyclohexanone and hydroxyacetophenone, which are putative substrates for such oxygenases (e.g [58,59]) were tested as carbon source for growth of strain DS-1^T^, as well as cycloalkanes (C_6_, C_8_, C_12_), however, none supported growth. The terpenoids camphor (for the involvement of a cytochrome-P450 oxygenase in the degradation pathway [60]) and geraniol, citronellol, linalool, menthol and eucalyptol (for the involvement of acyl-CoA interconversion enzymes in the degradation pathways) as substrates for growth were also tested negative.

In contrast to the high abundance of genes for aliphatic-hydrocarbon degradation, the genome contains few genes for aromatic-hydrocarbon degradation. One gene set for an aromatic-ring dioxygenase component (Plav_1761 and 1762; BenAB-type), three aromatic-ring monooxygenase component genes (Plav_1541 and 0131, MhpA-type; Plav_1785, HpaB-type), and three valid candidate genes for extradiol ring-cleavage dioxygenase (Plav_1539 [61] and 1787, BphC-type; Plav_0983, LigB-type) were predicted in the genome. Strain DS-1^T^ did not grow with benzoate, protocatechuate, phenylacetate, phenylpropionate, or phenylalanine and tyrosine as carbon source when tested.

Finally, *P. lavamentivorans* DS-1^T^ is predicted to store carbon in form of intracellular polyhydroxyalkanoate/butyrate (PHB) as its genome encodes a PHB-synthase (PhbC) gene (Plav_1129),  PHB-depolymerase (PhaZ) gene (Plav_0012), and PHB-synthesis repressor (PhaR) gene (Plav_1572).
